# Identifying potential drug targets and candidate drugs for COVID-19: biological networks and structural modeling approaches

**DOI:** 10.12688/f1000research.50850.3

**Published:** 2021-05-17

**Authors:** Gurudeeban Selvaraj, Satyavani Kaliamurthi, Gilles H. Peslherbe, Dong-Qing Wei

**Affiliations:** 1 Centre for Research in Molecular Modeling, Concordia University, Montreal, Quebec, H4B 1R6, Canada; 2 Centre of Interdisciplinary Science-Computational Life Sciences, College of Chemistry and Chemical Engineering,, Henan University of Technology, Zhengzhou, Henan, 450001, China; 3 The State Key Laboratory of Microbial Metabolism, College of Life Sciences and Biotechnology, Shanghai Jiao Tong University, Shanghai, Shanghai, 200240, China; 4 IASIA (International Association of Scientists in the Interdisciplinary Areas), 125 Boul. de Bromont, Quebec, J2L 2K7, Canada

**Keywords:** Biological network analysis, COVID-19, drug targets, ERBB4, growth factor receptor binding, Limma, protein-protein docking, SARS-CoV-2, signal transduction pathways, Walktrap algorithm, wortmannin

## Abstract

**Background: **Coronavirus (CoV) is an emerging human pathogen causing severe acute respiratory syndrome (SARS) around the world. Earlier identification of biomarkers for SARS can facilitate detection and reduce the mortality rate of the disease. Thus, by integrated network analysis and structural modeling approach, we aimed to explore the potential drug targets and the candidate drugs for coronavirus medicated SARS.

**Methods:** Differentially expression (DE) analysis of CoV infected host genes (HGs) expression profiles was conducted by using the Limma. Highly integrated DE-CoV-HGs were selected to construct the protein-protein interaction (PPI) network.

**Results:** Using the Walktrap algorithm highly interconnected modules include module 1 (202 nodes); module 2 (126 nodes) and module 3 (121 nodes) modules were retrieved from the PPI network. MYC, HDAC9, NCOA3, CEBPB, VEGFA, BCL3, SMAD3, SMURF1, KLHL12, CBL, ERBB4, and CRKL were identified as potential drug targets (PDTs), which are highly expressed in the human respiratory system after CoV infection. Functional terms growth factor receptor binding, c-type lectin receptor signaling, interleukin-1 mediated signaling, TAP dependent antigen processing and presentation of peptide antigen via MHC class I, stimulatory T cell receptor signaling, and innate immune response signaling pathways, signal transduction and cytokine immune signaling pathways were enriched in the modules. Protein-protein docking results demonstrated the strong binding affinity (-314.57 kcal/mol) of the ERBB4-3cLpro complex which was selected as a drug target. In addition, molecular dynamics simulations indicated the structural stability and flexibility of the ERBB4-3cLpro complex. Further, Wortmannin was proposed as a candidate drug to ERBB4 to control SARS-CoV-2 pathogenesis through inhibit receptor tyrosine kinase-dependent macropinocytosis, MAPK signaling, and NF-kb singling pathways that regulate host cell entry, replication, and modulation of the host immune system.

**Conclusion:** We conclude that CoV drug target “ERBB4” and candidate drug “Wortmannin” provide insights on the possible personalized therapeutics for emerging COVID-19.

## Introduction

Coronavirus (CoV) are the largest group of enveloped and single-stranded-ribonucleic acid (ssRNA) pathogens ^1^. The genome of CoV is typically larger (27-33Kb) than all other RNA viruses. Briefly, the genome contains an envelope protein, membrane protein, nucleoprotein, and spike protein which are responsible for capsid formation, assembly of virus particles and entry of the virus particle into the host ^2^. The CoVs are zoonotic in nature, i.e. transmitted between animals and humans. There were three unforgettable zoonotic infectious diseases outbreak caused by CoVs recorded in the past two decades. In the 20 ^th^ century (2002–2003), the foremost epidemic outbreak was originated in China by the severe acute respiratory syndrome (SARS) ^3^. Later, in the 21 ^st^ century (2012), the next epidemic outbreak happened in Saudi Arabia and the gulf countries with the Middle East respiratory syndrome (MERS) ^4^. In December 2019, the third epidemic outbreak was recorded in China, especially from Wuhan city through a novel severe acute respiratory syndrome CoV-2 (SARS-CoV-2) (COVID-19) ^5^. Paraskevis *et al.* (2020) ^2^ reported that the SARS-CoV-2 full-genome belongs to betacoronavirus, but it differs from the epidemic causing SARS and MERS ^6^. Moreover, the SARS-CoV-2 genome exhibits 96.3% similarity with the Bat-SARS viruses like CoVs. Among the various human CoVs (229E, NL63, HKU1, OC43, SARS, and MERS), SARS and MERS caused severe respiratory-related mortality rates of 10% and 37% respectively ^7^.

Recently, Huang *et al.* ^8^ reported that the most widespread symptoms of COVID-19 patients at the beginning of the disease's condition were respectively fever (98%), cough (76%), fatigue (44%) followed by sputum production (28%), headache (8%), blood-stained mucus (5%) and diarrhea (3%). Moreover, nearly one-fifth (19.2%) of individuals with COVID-19 were asymptomatic ^9^. COVID-19 positive cases showing the most common symptoms were cough hyposmia, sputum, and fever respectively ^9^. In this current pandemic situation, identification of novel biomarkers, which plays an effective role in prognosis and monitoring the status of the SARS-COV-2 disease condition, is necessary. A high-throughput oligonucleotide microarray profiling has been widely employed in measuring the expression of genes (>1000) level significantly. In addition, microarray facilitates the identification of biomarkers (diagnosis/therapeutic), classification of the disease condition, treatment options, and mechanism of action of the gene involved in pathogenesis ^10^. Biomarkers are identified through the determination of DEGs between the control and diseased/infected subjects, conversely finding the proteins that each biomarker is in connection with them, assisting to discover the key pathways related to the mechanism of the disease ^11– 13^. Many genes were involved in the pathogenesis, which make-up very complicated network systems. Coronavirus and influenza are pathogens causing severe respiratory illness to humans ^14^. Recent reports demonstrated that SP110, HERC5, SAMD9L, RTP4, ESPT11 genes were identified to control and improve the immune system and defense mechanism against influenza by network analysis ^15^. It has the supremacy to act as biomarkers and targets for pediatric influenza mediated therapy. A recent systematic review by 16 Kermali and his colleagues showed the increased level of biomarkers such as C-reactive protein, serum amyloid A, interleukin-6, lactate dehydrogenase, D-dimer, cardiac troponin and renal biomarkers (urea and creatinine) were higher, and low level of lymphocytes and platelet count were recorded in severe complicated COVID-19 patients than non-severe COVID-19 patients’ plasma and infected lung tissues.

**Figure 1.**
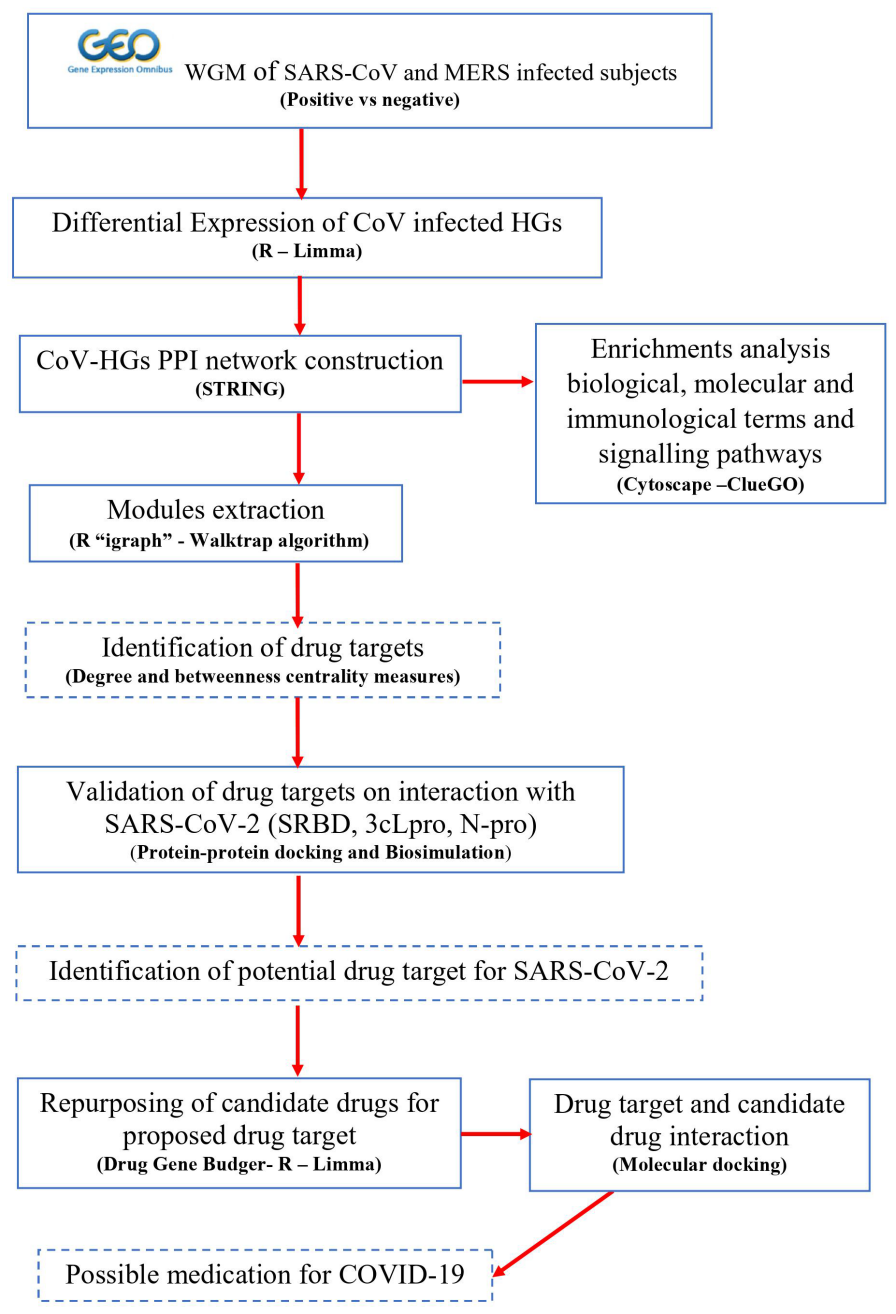
Schematic illustration of the study. WGM-whole genome microarray; SARS-CoV- ; MERS-; HGs-host genes; PPI-protein-protein interaction; SRBD-spike receptor binding domain; 3cLpro-3C-like protease; N pro-nucleocapsid protein;

Thus, the present study aimed to identify modules consists of the highly interconnected genes, which are involved in the pathogenesis of coronavirus medicated respiratory syndrome in humans. Microarray analysis of gene expression profiles is a standard and well-known method to identify potential targets and pathways ^17, 18^. Initially, we collected the gene expression profiles from the Gene Expression Omnibus (GEO) database. Then, we performed differential expression of CoV infected host genes (DE-CoV-HGs) using the Limma algorithm. The protein-protein interaction (PPI) network was constructed with DE-CoV-HGs and extracted different modules from the network using the Walktrap algorithm. Besides, the potential targets were predicted from the selected modules based on the degree and betweenness centrality measures, and determined their functional and pathway enrichment terms. The possible validation of potential targets interaction with proteins of SARS-CoV-2 was performed using protein-protein docking (PPD) and molecular dynamics (MD) simulations. Further, possible candidate drug for defined potential drug target (PDT) “ERBB4” using Drug Gene Budger. An insight into the study provides possible personalized therapeutic target and candidate drug for coronavirus medicated respiratory syndrome. Figure 1 illustrates the work flow of the study.

## Methods

### Data collection

The coronavirus infected host gene expression profiles were extracted from the GEO database ^19^. Agilent-014850 whole human genome microarray 4x44K G4112F platform was employed to GSE100509, GSE86529, GSE47962, GSE47961, GSE47960, GSE45042, GSE37827 and GSE33267 studies and one study GSE56677 which was performed based on Agilent-039494 SurePrint G3 human GE v2 8x60K microarray 039381 platform was included ^20– 24^. All the selected studies contain control and CoV infected human lung epithelial cells samples. The samples infected with other viruses were excluded. The sample details of different datasets are indicated in Table 1.

**Table 1.** List of microarray datasets used in this study.

Profile	Platform	Sample size (C vs I )	Pathogen	Reference
GSE47962	GPL6480	60 (33 vs 27)	SARS-CoV	20
GSE47961	GPL6480	60 (33 vs 27)	SARS-CoV	20
GSE47960	GPL6480	70 (38 vs 32)	SARS-CoV	20
GSE45042	GPL6480	32 (15 vs 17)	SARS-CoV	21, 24
GSE37827	GPL6480	60 (30 vs 30)	SARS-CoV	22
GSE33267	GPL6480	66 (33 vs 33)	SARS-CoV	22
GSE100509	GPL13497	50 (25vs 25)	MERS-CoV	24
GSE86529	GPL13497	50 (25vs 25)	MERS-CoV	24
GSE56677	GPL17077	33 (15 vs 18)	MERS-CoV	23

Note: C- Control; I- Infected; GPL13497 -Agilent-026652 Whole Human Genome Microarray 4x44K v2; GPL17077-Agilent-039494 SurePrint G3 Human GE v2 8x60K Microarray 039381; GPL6480 -Agilent-014850 Whole Human Genome Microarray 4x44K G4112F

### Differential expression of CoV infected host genes

Using NetworkAnalyst (NA) web interface, the tab-delimited text (.txt) files of selected datasets were given as an input for preprocessing, normalization, and probe identification ^25^. Variance stabilizing normalization (VSN) and quantile normalization was applied to reduce false-positive errors and equal distributions of datasets for statistical analysis ^26, 27^. R package “LIMMA” (Linear models for microarray analysis) of from the Bioconductor project were used to perform differential expression of CoV infected host genes ^28^. Log2 transformation, Benjamini and Hochberg and t-test were used to perform normalization and calculate false discovery rate (FDR; p<0.05) of samples ^29^.

### Construction of DE-CoV-HGs PPI network, modules and PDTs identification

We constructed the PPI network from identified DE-CoV-HGs using Search tool for retrieval of interacting genes/proteins (STRING) interactome ^30^. The highest confidence interaction score was set to 0.9, which reduces false positive interactions ^31^. The random walks (R package “ *igraph*”) was used to extract modules based on the Walktrap algorithm from the DE-CoV-HGs interaction network ^32^. It runs several short random walks within a group of nodes that are highly connected to detect small modules. From the modules, the hub genes (nodes) were identified using two different centrality measures “degree” and “betweenness” ^32^. The degree of the gene is the many connections it has to other genes. Genes with a high degree act as hubs within the network. The betweenness of a gene is the number of paths that pass through it when considering the pair-wise shortest paths between all genes in the network. A node that occurs between two dense clusters will have a high betweenness. In the present study, the PDTs of DE-CoV-HGs were identified based on the degree centrality and betweenness centrality measures.

### Pathway enrichment analysis

We have used ClueGO v2.5.3, which is a Cytoscape plugin for function and pathway enrichment analysis of DE-CoV-HGs ^33^. A list of PDTs were provided as input into ClueGO with select specific parameters like species such as-Homo sapiens; ID type-Entrez gene ID; enrichment functions -KEGG pathways for the analysis. Each enrichment was calculated based on the Bonferroni method (kappa score 0.96; *p* > 0.005). ImageGP was employed to visualize the results of functional enrichment analysis.

### Structural modeling approach to study host-SARS-CoV-2 proteins interaction and drug-target interaction

***Preparation of host and SARS-CoV-2 viral receptor.*** The FASTA sequence of selected proteins namely 3C-like protease (3cLpro), angiotensin I converting enzyme 2 (ACE2), B-cell lymphoma 3 (BCL3), casitas b-lineage lymphoma (CBL), CCAAT enhancer binding protein beta (CEBPB), CRK like proto-oncogene (CRKL), histone deacetylase 9 (HDAC9), Kelch-like protein 12 (KLHL12), v-myc myelocytomatosis viral oncogene (MYC), mothers against decapentaplegic homolog 3 (SMAD3), nucleocapsid protein (Npro), nuclear receptor coactivator 3 (NCOA3), receptor tyrosine-protein kinase erbB-4 (ERBB4), spike protein-receptor binding domain (SRBD), SMAD-specific E3 ubiquitin protein ligase 1 (SMURF1), ubiquitin C (UBC), and vascular endothelial growth factor A (VEGFA) were obtained from Uniprot for protein-protein docking studies ( Table 3) ^34^.

***Protein-protein docking.*** PPD was performed in the HDOCK web-interface using *ab initio* docking with a fast Fourier transform (FFT) based hierarchical algorithm ^35, 36^. The FASTA sequence of the selected protein (protein 1 and protein 2) was given as input. The server obtained and chosen one of the highest sequence coverage and similarity and with the highest resolution template automatically from PDB and then, the homology modeling was performed using MODELLER. Then, the corresponding structures constructed by superimposing the modeled protein structure on to the template, where 5,000steps of AMBER MD minimization was also performed to remove severe atomic clashes at the interfaces. The putative binding modes between the protein-1 and protein-2 are systematically samples based on the FFT algorithm. In total 4,392 binding modes are ranked depending on their cluster and binding energy scores. The final complex was selected with RMSD of two binding modes was below a cut off 5Å. RMSD was calculated based on the backbone atoms of the protein 2 ^36^.

$$\begin{array}{c} u_{xy}^{a+1}\;(z)=u_{xy}^{a}\;(z)+\Delta u_{xy}^{a}\;(z),\\ \Delta u_{xy}^{a}\;(z)=\frac{1}{2}a_{B}T\;\left[h_{xy}^{a}\;(z)-h_{xy}^{obs}\;(z)\right] \end{array}$$

Whereas, “ *a*” indicates as iterative step. “ *x*” and “ *y*” indicates the types of a pair of atoms in the protein 1 and protein 2. $h_{xy}^{obs}\;(z)$ is the pair distribution function for atom pair xy calculated from the ensemble of experimentally measured protein 1- protein 2 complex structures. $h_{xy}^{a}\;(z)$ is the pair distribution function calculated from the ensemble of the binding modes of the protein 1 – protein 2 complexes predicted with the trial potentials **{** $h_{xy}^{a}\;(z)$ **}** at the *a*-th step. $u_{xy}^{a+1}\;(z)$ are the improved potentials from $u_{xy}^{a}\;(z).\;a_{B}T$ . is the generality value set as 1 ^37^.

***Molecular dynamics simulations.*** The results of the PPD complex with the highest binding affinity were used to prepare input files for MD simulations. Solution builder in the CHARMM-GUI web interface was employed to solvate the complex with TIP3P and neutralized by potassium chloride (KCl) ions at the 0.15 mol-1 concentration under CHARMM36 force field ^38, 39^. The initial configuration of KCl ions is then estimated using short Monte Carlo simulations (2000 steps) through Coulombic and the van der Waals (vdW) interactions. The solvated complex was simulated by using Nanoscale molecular dynamics (NAMD) ^40^. Full system long-range Coulombic interactions were determined by the particle mesh Ewald (PME) method ^41^. The integrator parameters time was set to 2 fs/step. The simulations were executed in the constant pressure and temperature (NpT ensemble) with pressure bar (1) and temperature (303 K) through the Langevin dynamics with a damping coefficient of 1/ps. After 9×10 ^7^ steps of minimization, water molecules and ions were equilibrated for 2 ns around proteins, which were restrained using harmonic forces. MD simulation of selected drug target (ERBB4-3cLpro) was started from the last frame of restrained equilibration. The production was carried out for 150 ns.

### Identification of possible candidate drug for PDT and molecular docking

Drug Gene Budger a web-interface was employed to identify possible candidate drugs or small molecules in LINCS L1000 data that significantly regulated selected drug target (ERBB4) expression based on LIMMA algorithm ^42^. While entering the gene symbol of drug target, we could retrieve the following information such as log-transformed fold change, *p*-value and *q*-value for each potential small molecule. Small molecules/drugs that are liable for considerable overexpression and under expression of downregulated and upregulated drug target can increase the therapeutic action against COVID-19. The threshold *q* value was derived from the following equation

Q=X/Y=X/(X+Z)

Where X is the number of false discoveries and Z is the number of true discoveries. Y=X+Z is the number of rejected null hypotheses. The false discovery rate (FDR) is as follows

FDR=Qe=E[Q]

Where E [Q] is the expected value of Q. The drugs/small molecule have the highest log2FC value was selected for further protein-docking studies. The drug target ERBB4 sequence was retrieved from the Uniprot database (ID: Q15303) for three structure modeling. The protein structure modeling was performed in a Swiss modeling server. The structure of the candidate drug “wortmannin” was retrieved from the ZINC database (ID: ZINC1619592). Then, protein-ligand docking was performed to calculate the binding energy of the docked complex in the Swiss dock web-interface, which was developed based on the EADock dihedral space sampling (DSS) algorithm. The energy functions were calculated using the CHARMM force field. Each docking cluster was the output of 250 different sequential runs. The binding models (BM) having the most favorable energies were calculated by fast analytical continuum treatment of solvation (FACTS) and clustered. Binding modes were estimated through Full fitness and clustered. Then, the Full fitness of the clusters was estimated by averaging 30% of the most favorable effective energy of their elements.

The effective energy calculated by the following formula

$${\text{G}}_{\text{eff.eng}}={\text{E}}_{\text{intra}}{\;}^{\text{Ligand}}+{\text{E}}_{\text{intra}}{\;}^{\text{receptor}}+{\text{E}}_{\text{inter}}+{\text{G}}_{\text{elec.solv}}+\text{σ}\times \text{SASA}$$

Whereas, E _intra_^Ligand^, E _intra_^receptor^ are the internal energies of ligand and receptor; E _inter_ is interaction energy between the receptor and ligand; σ value (0.0072 kcal/mol); SASA is the solvent accessible surface area. The results were visualized in Maestro Visualizer. All the supporting data associated with this study freely available ^43^.

## Results

### Differentially expressed -CoV-HGs

In total, 1,968 DE-CoV-HGs in human lung epithelial cells were obtained from the initial analysis. To obtain better results of biomarker identification, we have selected 315 overexpressed and 112 under expressed DE-CoV-HGs based on the log-fold changes -1>log2FC>1. Table 2 illustrates the top 10 DE-CoV-HGs, their description, and their fold change and *p* value. In addition, one of the key receptor human angiotensin I converting enzyme 2 (ACE2) is responsible for primary infection. But, the expression level of ACE2 found to be less significant (log2FC<1) in the infected cells of the selected datasets. Thus, ACE2 excluded in the PPI network analysis. The complete list of differentially expressed genes included in appendix ^43^).

**Table 2.** Top 10 differentially expressed CoV-HGs.

Gene symbol	Name	Log2FC	*p* value
**Overexpressed genes**			
CALCA	Calcitonin related polypeptide alpha	3.149	2.78×10 ^-12^
GALNTL2	UDP-N-acetyl-alpha-D-galactosamine: polypeptide N-acetylgalactosaminyltransferase-like 2	2.581	5.46×10 ^-14^
SPIN4	Spindlin family, member 4	2.362	7.50×10 ^-16^
ARL4A	ADP-ribosylation factor-like 4A	2.336	1.84×10 ^-19^
CCL14	Chemokine (C-C motif) ligand 14	2.255	4.08×10 ^-14^
PGM5	Phosphoglucomutase 5	2.229	7.28×10 ^-14^
OTUD3	OTU deubiquitinase 3	2.213	6.63×10 ^-16^
GIMAP7	GTPase, IMAP family member 7	2.202	2.52×10 ^-15^
YAP1	Yes associated protein 1	2.178	1.22×10 ^-04^
CLU	Clusterin	2.155	6.87×10 ^-15^
**Under expressed genes**			
SSX2	Synovial sarcoma, X breakpoint 2	-8.803	4.39×10 ^-20^
FBXL8	F-box and leucine-rich repeat protein 8	-7.468	3.48×10 ^-14^
NEURL3	Neutralized homolog 3 (Drosophila) pseudo gene	-5.084	5.86×10 ^-15^
TSPAN18	Tetraspanin 18	-4.866	2.07×10 ^-04^
FXYD3	FXYD domain containing ion transport regulator 3	-3.949	9.70×10 ^-13^
CELF4	CUGBP, Elav-like family member 4	-3.616	2.95×10 ^-04^
SLC12A1	Solute carrier family 12 member 1	-3.443	2.29×10 ^-04^
BNC2	Basonuclin 2	-3.412	2.51×10 ^-04^
KLF10	Kruppel-like factor 10	-3.386	5.91×10 ^-22^
TNFAIP3	Tumor necrosis factor, alpha-induced protein 3	-3.314	1.68×10 ^-16^

**Table 3.** List of drug targets and PPD interaction with SARS-CoV-2 proteins.

Potential target gene encoded host target proteins	Cellular components	Degree centrality	Betweenness centrality	Log2 FC	Uniprot ID	Reference PDB ID	PPD binding affinity (kcal/mol)		
							SRBD Uniprot ID: P0DTC2	3cLpro Uniprot ID: P0DTD1	N-protein Uniprot ID: P0DTC9
Bcl3	N, NP, CP	16	1364.72	-1.621	P20749	1K1A	-265.8	-288.55	-244.73
CBL	N, CP, C, PM	81	6405.31	1.046	P22681	5HKX	-285.74	-257.97	-244.71
CEBPB	N, NP, CP	28	2647.13	-1.869	P17676	3A5T	-235.81	-220.39	-227.41
CRKL	EN, C, EX	32	1158.21	1.081	P46108	2EYZ	-302.98	-273.78	-276.28
ERBB4 *	**N, NP, M, C, PM**	**34**	**3037.11**	**-2.052**	Q15303	3U9U	**-289.18**	**-280.6**	**-281.11**
HDAC9	N, NP, CP	46	6027.63	1.027	Q9UKV0	5JI5	-286.06	-262.5	-261.77
KLHL12	GM, C	15	538.67	1.023	Q53G59	2VPJ	-290.49	-289.48	-279.35
MYC	N, NP, M, C	89	12884.19	-2.021	P01106	4ATI	-238.61	-244.15	-197.38
NCOA3	N, CP, EX	30	3580.11	1.059	Q9Y6Q9	4F3L	-265.44	-281.1	-239.99
SMAD3	N, CP, C, PM	69	5195.05	-1.353	P84022	1KHX	-288.66	-271.12	-254.11
SMURF1	N, CP, M, C, PM	48	3655.56	-1.163	Q9HCE7	5HPL	-269.9	-241.05	-233.53
VEGFA	C, PM	9	1123.93	0	P15692	2VWE	-234.15	-230.48	-243.59
UBC	N, NP, CP, C, PM	21	2832.51	-1.905	P0CG48	3RT3	-240.33	-218.27	-222.44
ACE	PM	-	-	-	Q9BYF1	1R42	-291.07	-	-

**Note:** * denotes ERBB4 selected as drug target; 3cLpro -3C-like protease; ACE2-angiotensin I converting enzyme 2; Bcl3-B-cell lymphoma 3; CBL- casitas b-lineage lymphoma; CEBPB- CCAAT enhancer binding protein beta; CRKL- CRK like proto-oncogene; C-cytosol; CP-cytoplasm; ERBB4- receptor tyrosine-protein kinase erbB-4; EN-Endosome; EX-exosome; FC -fold change; HDAC9- histone deacetylase 9; KLHL12- Kelch-like protein 12; M-mitochondria; MYC- v-myc myelocytomatosis viral oncogene; GM-golgi membrane; N protein- nucleocapsid protein; N-Nucleus; NP-nucleoplasm; PM-plasma membrane; PPD- protein-protein docking; SMAD3-mothers against decapentaplegic homolog 3; NCOA3- nuclear receptor coactivator 3; SMURF1-SMAD-specific E3 ubiquitin protein ligase 1; SRBD- spike protein-receptor binding domain; UBC-ubiquitin C; VEGFA-vascular endothelial growth factor A; FC-Fold change

### Modules and potential drug targets identification

**Figure 2.**
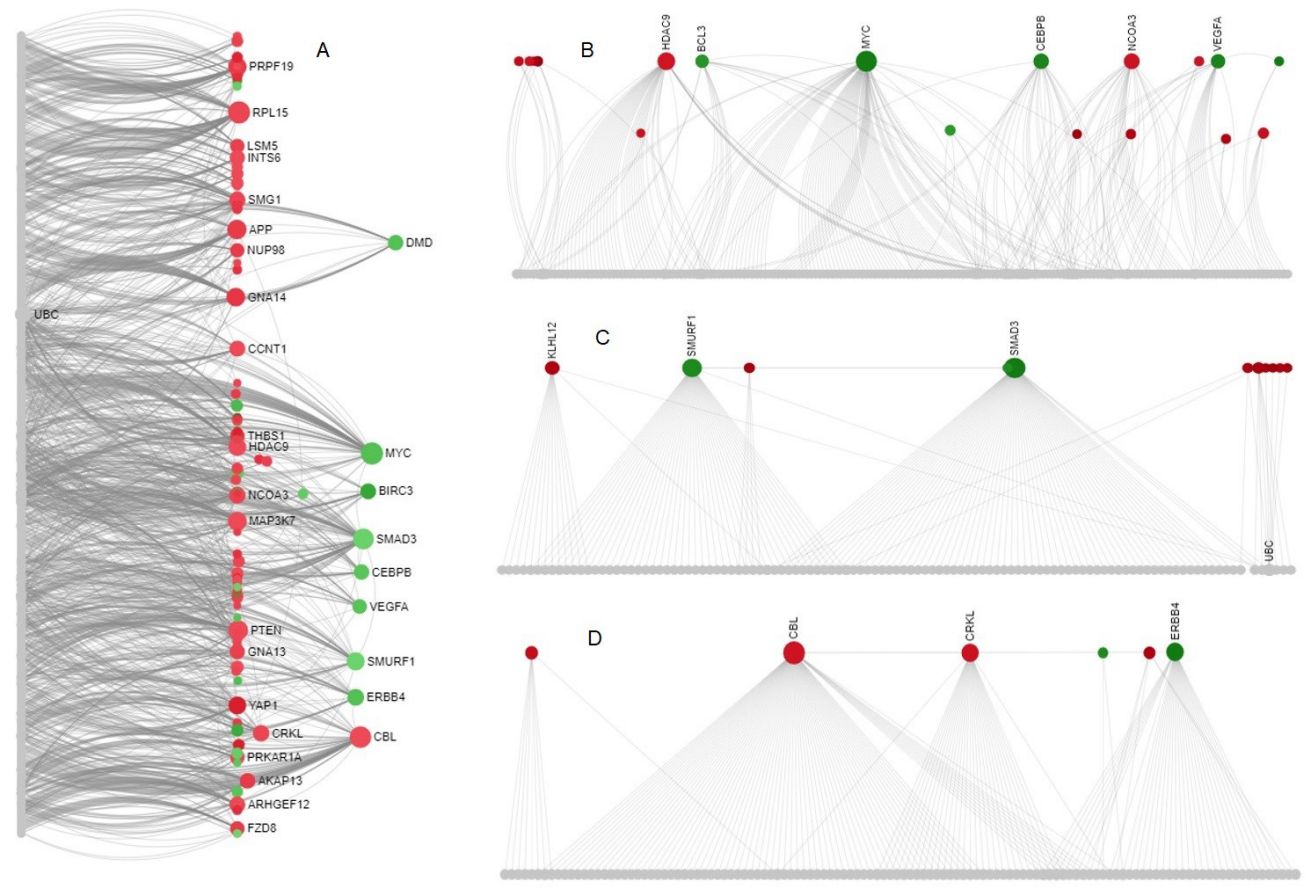
PPI network and Walk trap modules from DE-CoV-HGs. ( **A**) PPI network with highly interconnected nodes; ( **B**) Module-1; ( **C**) Module-2; ( **D**) Module-3. Overexpressed genes are represented in green color, under-expressed genes in red color, interconnected nodes in gray color.

PPI network was constructed using 427 DE-CoV-HGs ( Figure 1a). The major subnetwork contains 531 edges with an average local clustering coefficient 0.353 and the PPI enrichment *p*-value was 2.36 ×10-3. Then, using the Walktrap algorithm, 47 modules were retrieved from the subnetwork. Among them, highly interconnected modules includes module 1 (No. of nodes=202; p=5.20×10 ^-32^); module 2 (No. of nodes=126; p=1.90×10 ^-28^) and module 3 (No. of nodes=121; p=3.13×10 ^-26^) was employed to identify the PDTs ( Figure 2b–d). Table 3 demonstrated that list of PDTs identified from different modules based on the degree and betweenness centrality measures. BCL3, CBL, CEBPB, CRKL, HDAC9, KLHL12, MYC, SMAD3, NCOA3, ERBB4, SMURF1, UBC, VEGFA were identified as PDTs which are highly expressed in human cells after CoV infection.

### Functional and pathway enrichments of PPI network and PDTs

**Figure 3.**
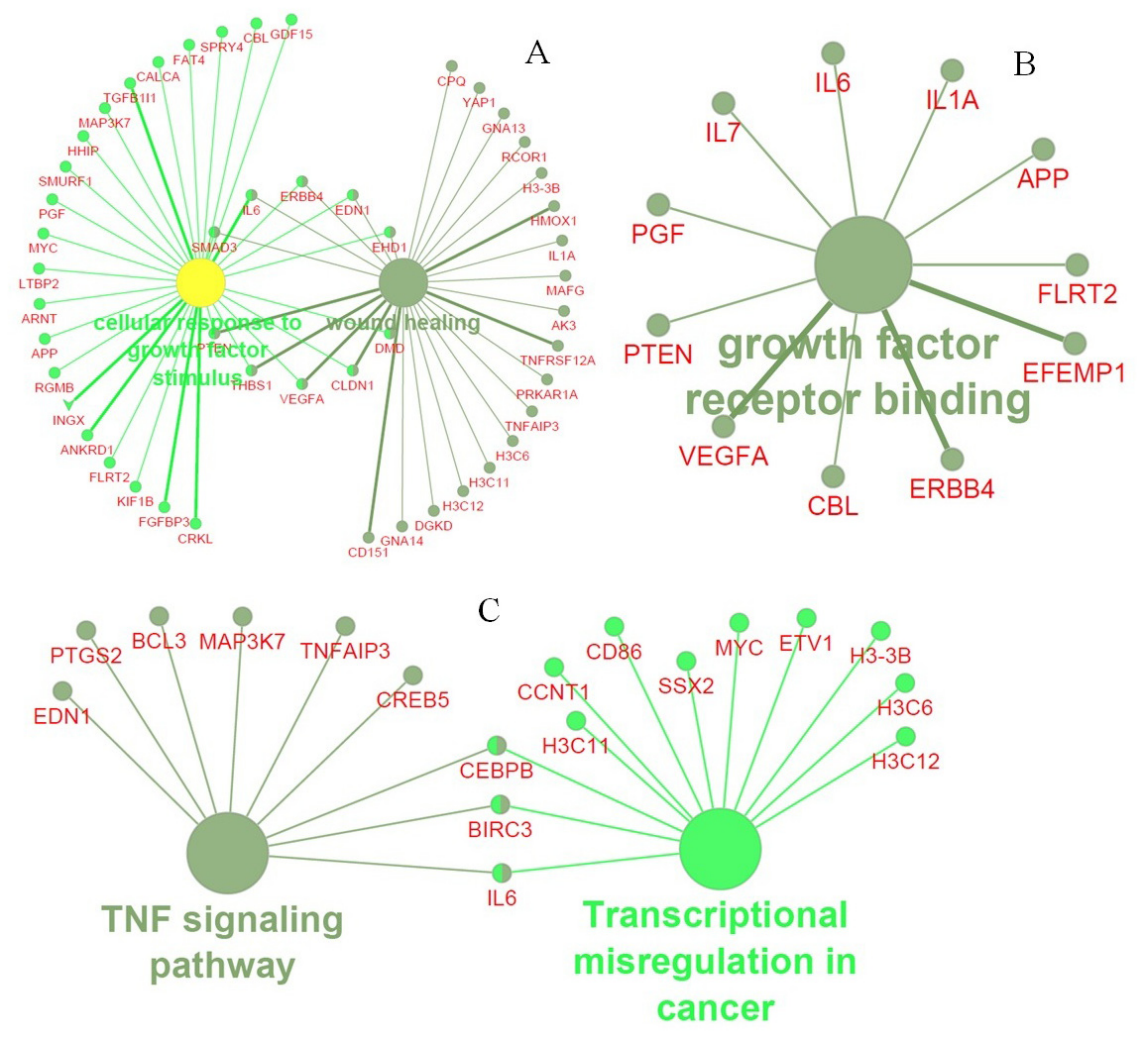
GO and Pathway enrichments of the PPI network. ( **A**) Biological process term; ( **B**) Molecular function term; ( **C**) Pathway enrichment terms of PPI network.

**Figure 4.**
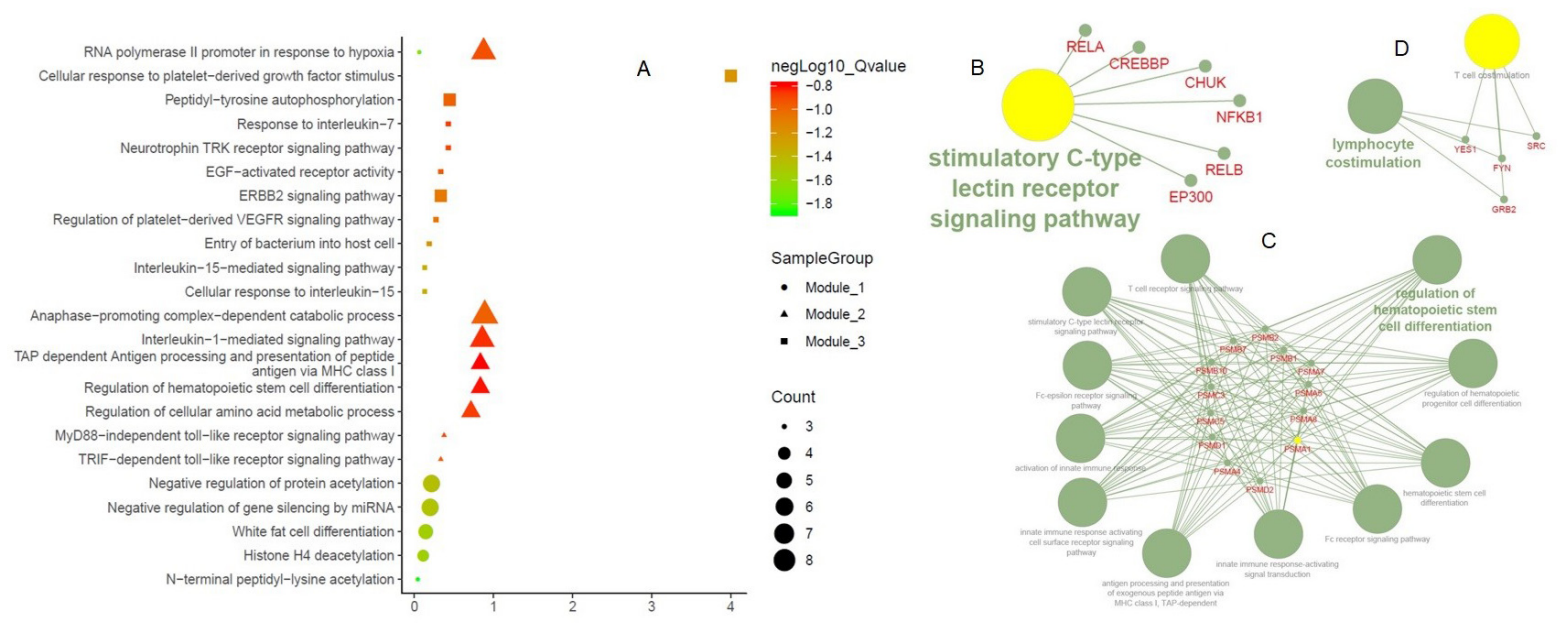
Functional enrichments of the Walk trap modules. ( **A**) Biological process terms of three different modules; Immunological terms of ( **B**) Module-1; ( **C**) Module-2; ( **D**) Module-3.

**Figure 5.**
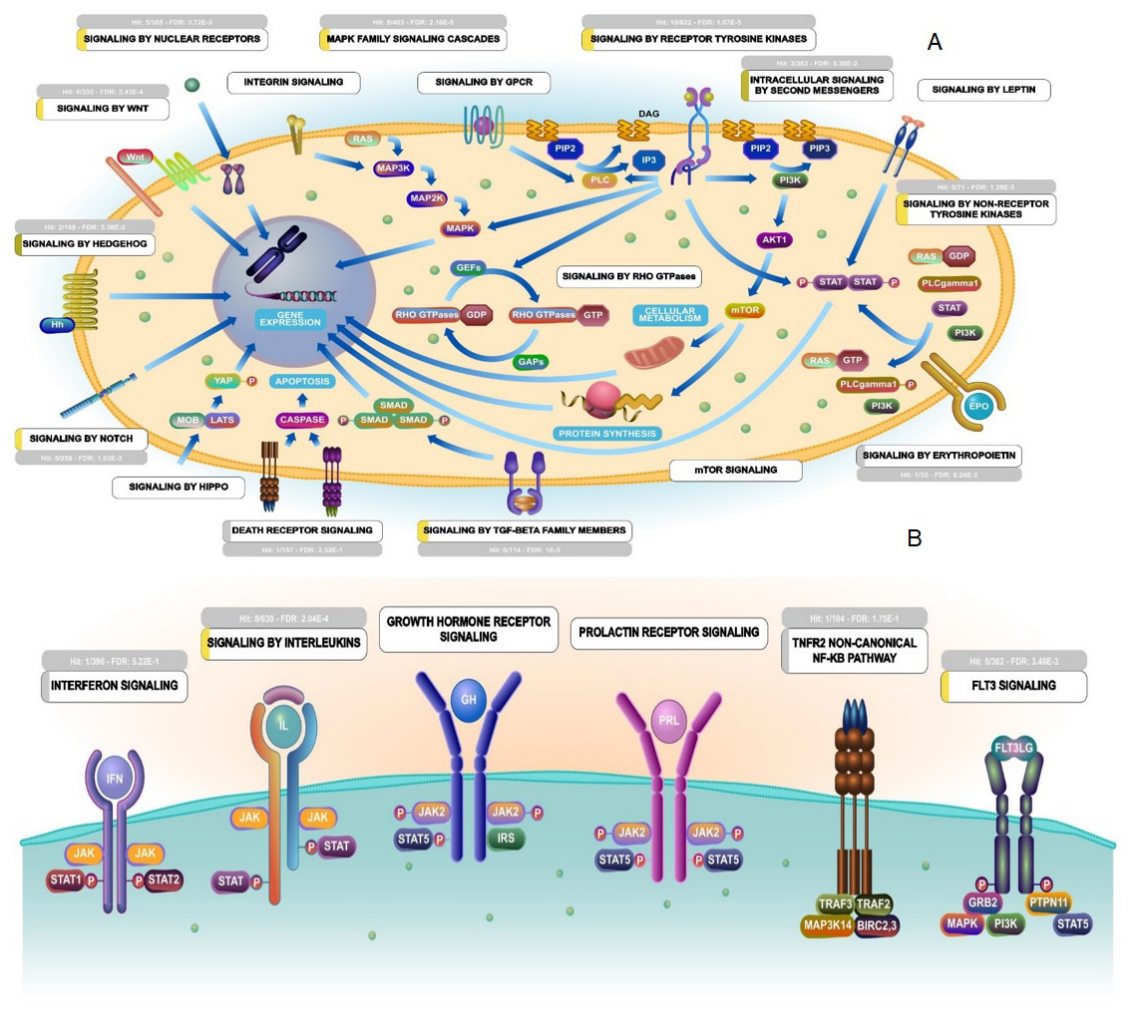
Pathways enrichment of Walktrap modules. ( **A**) Signal transduction pathways; ( **B**). Cytokine immune signaling pathways. The figures are retrieved from Reactome pathway analyzer using PTGs.

Many important gene ontology terms and pathways were identified from the PPI network namely, cellular response to growth factor stimulus, wound healing as a biological process term; growth factor receptor binding as a molecular function term; and TNF signaling pathway and transcriptional mis-regulation in cancer as a pathway enrichment terms ( Figure 3). Moreover, the biological process term that is directly or indirectly related to the viral life cycle and human immune system includes RNA polymerase II promoter in response to hypoxia, peptidyl-tyrosine auto-phosphorylation, ERBB2 signaling pathway, anaphase-promoting complex dependent catabolic process, interleukin-1 mediated signaling pathway, TAP dependent antigen processing and presentation of peptide antigen via MHC class I, regulation of cellular amino acid and metabolic process, negative regulation of protein acetylation, negative regulation of gene silencing by miRNA, white fat cell differentiation and histone H4 deacetylation enriched in three different modules. Besides, stimulatory c-type lectin receptor signaling pathway (module 1), regulation of hematopoietic stem cell differentiation, T cell receptor signaling, innate immune response signaling and Fc-epsilon receptor signaling pathway (module 2), lymphocyte co-stimulation (module 3) were highly enriched immunological terms of three different modules ( Figure 4). Further, pathways enrichment of modules demonstrated that numerous signal transduction pathways and cytokine immune signaling pathways were illustrated in Figure 5.

### Molecular interaction of host drug targets with SARS-CoV-2 protein

**Figure 6.**
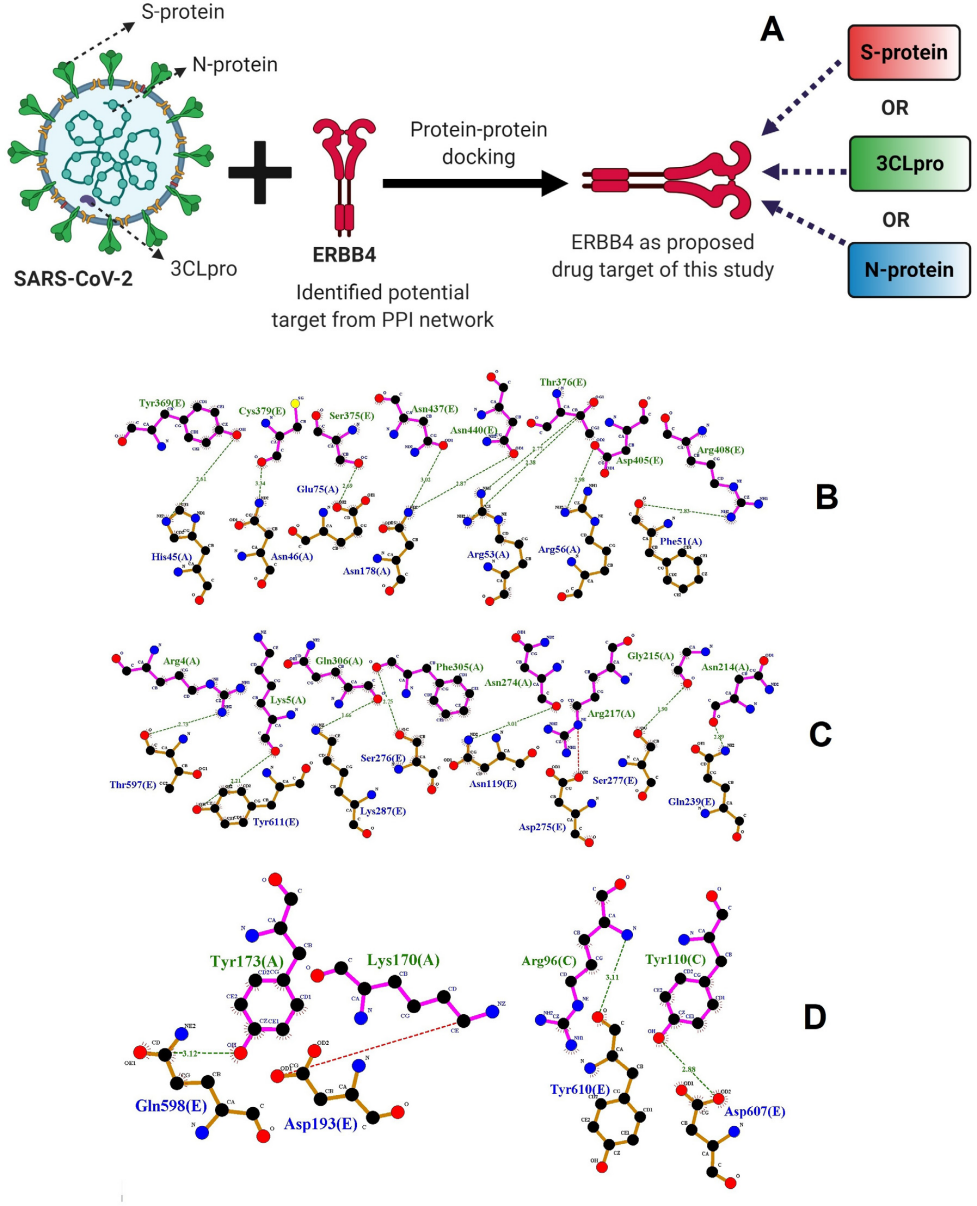
Possible action and PPD of drug target ERBB4 with SARS-CoV-2 proteins. ( **A**) Possible pathological action of SRBD, 3cLpro, and N-protein of SARS-CoV-2 with “ERBB4”. Results of PPD of ( **B**) ERBB4-SRBD; ( **C**) ERBB4-3cLpro; and ( **D**) ERBB4-N-protein complex by using LigPlot. The interacting residues of ERBB4 (magenta) are labelled in green color and SARS-CoV-2 protein structures (brown) are labelled in blue color. The interacting hydrogen bonds indicated in green color.

***Protein-protein docking interaction.*** The potential target genes encoding proteins are defined as drug targets to control the pathogenesis of SARS-CoV-2. The drug targets regulated (overexpressed or under-expressed) by SARS-CoV-2 during pathogenesis in the host cytoplasm and different cellular components. Accordingly, PPD of SRBD, 3cLpro, and N-protein of SARS-CoV-2 with proposed drug targets happened by different biological signaling pathways. Thus, we hypothesized, proposed drug targets may control the pathogenesis of SARS-CoV-2 through the respective signaling pathways. The possible molecular interaction of host drug targets with SRBD, 3cLpro, and N-protein of SARS-CoV-2 was determined by PPD method. In the present study, we performed protein-protein rigid-body docking, which treats both proteins as rigid and discovers only six degrees of translational and rotational freedom. It excludes any kind of flexibility. Drug targets include HDAC9, SRBD, SMURF1, SRBD, CBL, ERBB4, BCL3, CEBPB, CRKL, KLHL12, MYC, NCOA3, SMAD3, UBC, and VEGFA were in the human respiratory system, which was possibly able to interact with the S-RBD, 3cLpro, and N - protein of SARS-CoV-2 and regulate the viral pathogenesis through replication and development. PPD binding affinities were illustrated in Table 3. Among the drug targets, ERBB4 demonstrated showed high binding affinity -260.46, -314.57 and -291.72 kcal/mol with SRBD, 3cLpro and N-protein of SARS-CoV-2 respectively. The binding affinity of ERBB4 and KLHL12 with SARS-CoV-2 targets was almost similar, but both proteins differ in the level of expression in infected cells. In general, the potential drug target selection is based on the log2 fold change (i.e. -2>log2FC<2). ERBB4 is highly under-expressed (i.e. log2FC=-2.052) compared to other identified drug targets. Thus “ERBB4” was selected as potential drug target and used for candidate drug selection study. Hydrogen bond forming residues between the ERBB4 and SRBD, 3cLpro, and N-protein of SARS-CoV-2 complexes represented in Figure 6. 

**Figure 7.**
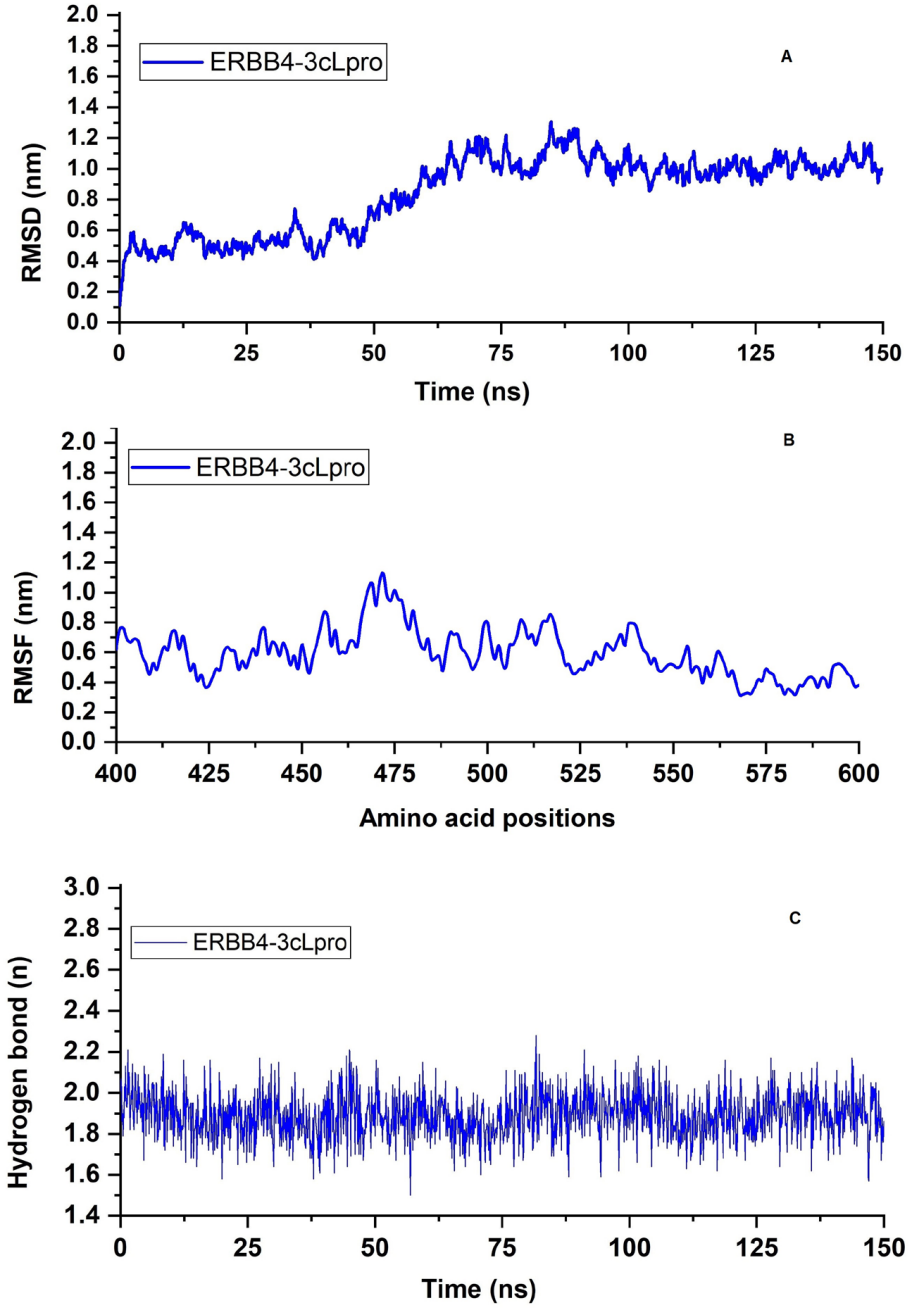
MD simulation of ERBB4-3cLpro complex. ( **A**) NAMD trajectories of RMSD; ( **B**) RMSF; ( **C**) Total number of intermolecular hydrogen bond formation trajectories of ERBB4-3cLpro complex.

***MD simulation of ERBB4-3cLpro complex.*** MD simulations demonstrated the stability of the ERBB4-3cLpro complex. The structure and dynamic properties of the complex were followed by monitoring the backbone Cα root mean square deviation (RMSD) from the initial structure during the simulation period (150ns). The RMSD of the ERBB4-3cLpro complex gradually increased for the first 95 ns before steadily oscillating around a value of ~0.85 nm, indicative of the ERBB4-3cLpro complex stability over the simulation timescale ( Figure 7a). The root mean square fluctuation (RMSF) reflects the fluctuations in the positions of the ERBB4-3cLpro complex residues. Briefly, notable fluctuations have been observed in the multiple furin-like cysteine-rich domains, a tyrosine kinase domain, and a phosphatidylinositol-3 kinase binding site of ERBB4. However, the other amino acid residues in the PDZ binding domain (amino acid range 400–600) were not deviating more ( Figure 7b). This indicates the reason for the stable RMSD plot of the ERBB4-3cLpro complex. The strong hydrogen bonding interaction (average ~1.89) between ERBB4 and 3cLpro observed throughout the 150ns of MD simulation ( Figure 7c).

### Drug candidate for proposed drug target

**Figure 8.**
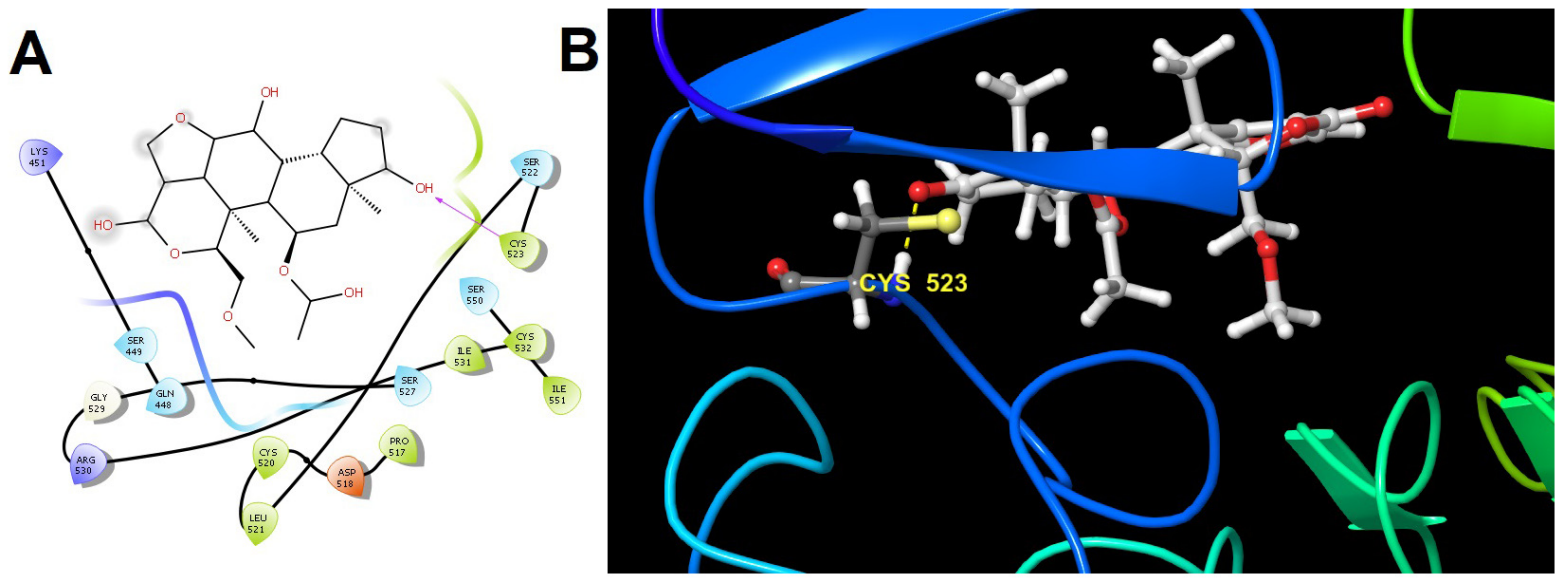
Molecular interactions of Wortmannin within the catalytic pocket residues of receptor ERBB4. ( **A**) Two dimensional (hydrogen bond formation between Cys523 residue of ERBB4 and OH atom of Wortmannin showed in magenta line) and ( **B**) three-dimensional interaction (hydrogen bond formation between Cys523 residue of protein-ligand complex showed in yellow color dotted line).

The results of Drug Gene Budger demonstrated that Wortmannin ( *q*- value = 3.31×10 ^-21^; log2 Fold change = 2.997; Specificity = 1.09×10 ^-04^) was identified as the candidate drug for the proposed target ERBB4. The ERBB4 was downregulated after the coronavirus infection that was then upregulated by the possible use of the Wortmannin as candidate drugs, which may inhibit the pathological cycle and development of SARS-CoV-2 in the human hosts. 30 different protein-ligand cluster of binding modes were obtained after molecular docking. Among them, the binding mode of cluster 1 indicated that the ERBB4-Wortmannin complex has the energy in term of full fitness -2563.6987 kcal/mol and Gibbs free energy ∆G was -7.93 kcal/mol ( Figure 8).

## Discussion

The discovery of biomarkers from gene-expression profiles as well as key functions in a pathogenic-related pandemic disease like COVID-19 is of consequence for diagnosis, drug development, and monitoring of disease progression. Network analysis provides new insights into the biological and cellular organization. The single gene can be involved in many biological functions and regulated different genes at different times ^44^. Thus, biomarkers from gene-expression profiles of coronavirus infected subjects have the potential to be employed as drug-targets for COVID-19. Therefore, the present study aimed to identify drug targets from modules containing highly interconnected potential target genes which involve in the pandemic coronavirus pathogenesis. DEGs analysis demonstrated that 315 overexpressed and 112 under expressed genes between normal and CoV-infected subjects. Besides, the potential target genes namely MYC, HDAC9, NCOA3, CEBPB, VEGFA, BCL3, SMAD3, SMURF1, KLHL12, CBL, ERBB4, and CRKL were identified from Walktrap modules, which are significantly associated with the coronavirus infection. Then, the potential target genes encoding proteins employed as PDTs (receptor) for further analysis. Thus, we discussed the possible association of PDTs with pathogenesis of SARS-CoV-2.

MYC regulates numerous cellular functions including cell activation, differentiation, cell cycle progress, transformation, and apoptosis in virus (HIV, hepatitis C, influenza, and Epstein-Barr virus (EBV)) infected host cells ^45– 47^. In addition, mutation of MYC can disturb human B cells to proliferate indefinitely ^48, 49^. Further, influenza A virus infection dysregulates the expression of miRNA-22 and induces CD147 in asthmatics through the MYC transcription factor ^50^. Moreover, overexpression of the MYC gene associated with PHACTR3 and E2F4 mutation in NSCLC is considered as a potential biomarker of NSCLC and its specific subtypes ^51^. This information suggesting that targeting MYC for COVID-19 or CoV mediated respiratory disease has a potential role in the SARS-CoV-2 pathogenesis. SMAD3 is a transcriptional regulator and plays as a mediator of the cellular signals initiated by the transforming growth factor-beta (TGF-β) superfamily of cytokines, which control proliferation, differentiation, and apoptosis. It is a complex regulator in adipose physiology and the pathogenesis of chronic obstructive pulmonary disease, diabetes, inflammatory disease, and obesity ^52, 53^. In addition, TGF-β/SMAD3-induced repression of target genes, it required repression of the MYC gene. Moreover, SMAD3 deficiency induces inflammatory abnormal bulge on aortic in angiotensin II-infused animal models through the activation of nitric oxide synthases ^54^. This information suggesting that under-expressed SMAD3 in CoV mediated respiratory disease has a prospective role in the pathogenesis of SARS-CoV-2. CBL is a proto-oncogene, encoding one of the ubiquitin ligase protein CBL involving in cell signaling, and causes a mutation in the lung, colon, endometrial adenocarcinoma, cutaneous, and breast cancer ^55^. In addition, CBL is a potential gatekeeper of immune activation through its function as a non-redundant negative regulator of immune activation. Further ^56^, reported that targeting CBL and lipid rafts have the potential to block Kaposi's sarcoma-associated herpesvirus infection of endothelial cells. Moreover, CBL was identified as modulators of immature dendritic cell (DC) activation. The deficiency of CBL in DC upregulates toll-like receptors and inducing pro-inflammatory cytokines and chemokines ^57^. Then, reduced expression of CBL in CD4+T cells is reported in several autoimmune diseases namely asthma, lupus erythematodes, multiple sclerosis, and type 1 diabetes ^58^. This information is proposing that overexpressed CBL in CoV infected subjects have a prospective role in the host immunological system.

HDAC9 belongs to class II histone deacetylases, and plays a crucial role in regulating adipocyte and myocyte differentiation, and cardiac muscle development. It generally applies its function in the nucleus, and thus its activity is inhibited due to cytoplasmic retention ^59^. In addition, HDAC9 transcription is observed significantly high in CD4+ T-cells from lupus subjects compared with healthy subjects ^60^. NCOA3 is a transcriptional coactivator that directly binds different nuclear receptors and stimulates hormone-dependent transcriptional activities. In addition, it plays a central role in the remodeling of chromatin, histone acetyltransferase activity, and NF-kB pathway ^61, 62^. It is effectively used as a biomarker in breast and hepatocellular cancer ^63, 64^. VEGFA is a signaling protein produced by different types of cells includes macrophages, tumor cells, platelets, keratinocytes, and renal mesangial cells, which play an active role in endothelial cell functions including the formation of bones, and the angiogenesis and hematopoiesis processes. Moreover, the VEGF-A isoform enhanced the entry of the virus into the host cells through the activation of this Akt pathway ^65^. Furthermore, SARS coronavirus can diffuse alveolar damage with changeable degrees of acute edema and hyaline membranes, organization, fibrosis, macrophagic infiltration, multinuclear giant cells, atypical reactive pneumocytes, and vascular injury ^66, 67^. This literature information suggested that accepting the relationship between coronavirus mediated respiratory disease and VEGF signaling biomarkers will cover the way to design targeted and effective therapeutic approaches for emerging COVID-19. BCL3 is a proto-oncogene that resides in the nucleus and plays a key role in the regulation of inflammatory response through the transcription of genes dependent on the NF-κB. In the context of respiratory syncytial virus (RSV) infection, the BCL-3 changes the status of histone acetylation and transcription factors on activated inflammatory (chemokines) promoters ^68^. In another study, the BCL-2 gene was significantly up-regulated in PBMC samples of SARS patients (5 fold) than compared to healthy individuals ^69^. BCL-3 prevents the inflammation in injured lungs through pinning-down the emergency granulopoiesis process ^70^. The SARS-CoV open reading frame 7a (SARS-CoV-7a) protein participated in various pathogenesis processes including interaction with host protein and inhibiting their synthesis, promoting apoptosis, and activation of p38MAPK in the host system ^71^. Further, the overexpression of BcL-xL protein in transected Jurkat T-cells significantly block the induction of apoptosis by SARS-CoV-7a protein ^72^.

For the biological process, we determined that cellular response to growth factor stimulus wound healing was the key function of the PPI network. The top five functional terms were RNA polymerase II promoter in response to hypoxia, peptidyl-tyrosine auto-phosphorylation, ERBB2 signaling pathway, anaphase-promoting complex dependent catabolic process, interleukin-1 mediated signaling pathway of the modules. For molecular function, growth factor receptor binding is the major term. Growth factor receptor (GFR) mostly belongs to tyrosine kinase receptors or serine-threonine kinases. The cytoplasmic domain of GFR acts as an enzyme or binds to another protein and forms a complex that acts as an enzyme. In addition, binding of GFR leads to phosphorylation of tyrosine residue and transmitting cell signals within the cell ^73^. After, viral infection, the ligand (viral proteins) binds to the GFR through cell signaling, phosphorylation, and rearrangement and activates different downstream signaling that enhances cell survival, proliferation, angiogenesis, and endocytosis ^74^. Moreover, a recent report demonstrated that GFR as a potential target for drug repurposing against emerging viral diseases such as COVID‐19 ^75, 76^.

Moreover, we also expected to determine which immunological terms linked with coronavirus pathogenesis; namely stimulatory c-type lectin receptor (CLR) signaling pathway, regulation of hematopoietic stem cell differentiation, T cell receptor signaling, innate immune response signaling, and Fc-epsilon receptor signaling pathway, lymphocyte co-stimulation were highly enriched immunological terms of three different modules. The carbohydrate-recognition domains of CLRs are important pattern recognition receptors to identify viral pathogens and induce antiviral innate immune responses and T helper differentiation ^77^. Further, reports suggested that the activation of CLRs and RIG-I-like receptors enhances pro-inflammatory response in MERS coronavirus-infected macrophages ^78^.

For KEGG pathways, we identified 12 important pathways that might have an essential role in the pathogenesis and human immune system. Eight different signal transduction pathways include nuclear receptor, MAPK, and WNT signaling pathway, receptor tyrosine kinase, intracellular signaling by second messenger, TGF-β, Notch and Hedgehog signaling pathway; four key cytokine immune signaling pathways namely signaling of interleukins, TNFR2-NF-kB pathway, FLT3, and interferon signaling pathways were identified using the hub-genes. Many studies reported that MAPK, WNT, receptor tyrosine kinase, intracellular signaling by the second messenger, TGF-β, Notch, and Hedgehog signaling pathway regulated in viral pathogenesis ^79, 80^. As we stated above, overexpression of HDAC9, NCOA3, KLHL12, CBL, and CRKL and under expression of MYC, CEBPB, VEGFA, BCL3, SMAD3, SMURF1, and ERBB4 may be involved in the regulation of pathways in signal transduction and cytokine immune signaling pathways. It might act as a novel drug target of coronavirus infected patients and plays a key role to improve host immune system.

Many biological functions, like cell signaling, cellular metabolism, enzyme inhibition and antibody-antigen recognition, have essential molecular interaction whether receptor-receptor or receptor-drug interaction. These molecular interactions frequently lead to form protein-protein or protein-drug complexes to carry out molecular functions ^81^. Unfortunately, there are insufficient gene expression profiles of SARS-CoV-2 infected patients in the GEO database. Thus, in the present study, we have validated that drug targets identified from SARS-CoV-1 infected patients can have the possible interaction with SRBD, 3cLpro and N-protein of SARS-CoV-2 to define possible drug targets. Understanding the binding mode and affinity between PTs encoded host proteins and target proteins of SARS-CoV-2 requires tertiary structure of these proteins. However, it is generally difficult and expensive to obtain complex structures by nuclear magnetic resonance spectroscopy and X-ray crystallography. Thus, protein-protein docking is an important method for understanding interactions of drug targets with SRBD, 3cLpro and N-protein of SARS-CoV-2. In the present study, the protein-protein docking results demonstrated that drug-targets with three different protein of SARS-CoV-2 showed the highest interaction energies Hydrogen bond forming residues between the protein complexes. Among them, the highly under-expressed gene ERBB4 ( Table 3) encoding drug target “ERBB4” demonstrated high docking interaction energies -260.46, -314.57 and -291.72 kcal/mol with SRBD, 3cLpro and N-protein of SARS-CoV-2 respectively, which was selected for further candidate drug selection studies. 

### Precision based therapeutic countermeasures for COVID-19 patients

**Figure 9.**
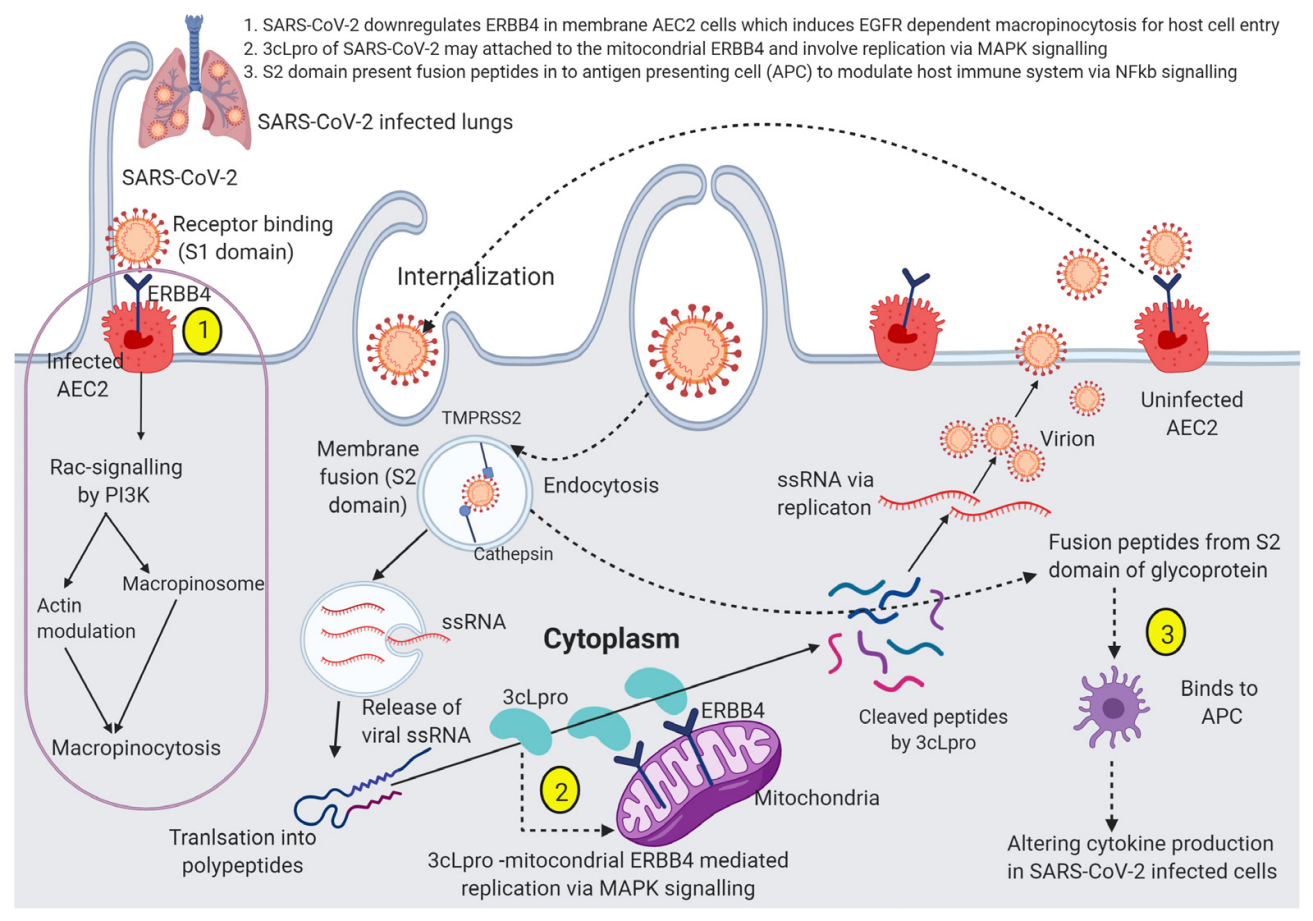
Hypothesized mechanism of action of downregulated ERBB4 in SARS-CoV-2 infected cells. There are three possible mechanisms (1) SARS-CoV-2 an enveloped single-strand RNA virus, virions are internalized probably by receptor tyrosine kinase (EGFR) family receptor ERBB4 dependent macropinocytosis like SARS-CoV-1 (the process of macropinocytosis indicated in magenta circle). The cellular entry of SARS-CoV-2 involves binding to ERBB4 expressing AEC2 in the plasma membrane, macropinocytosis, and cathepsin based cleavage of the spike glycoproteins. The host transmembrane serine protease 2 (TMPRSS2) for spike priming and cathepsin cleaves spike glycoprotein S1 domain, permits fusion with endosomal membranes and release virions genome into the host cytoplasm; (2) Single-strand RNA translated polypeptides cleaved by the 3cLpro of SARS-CoV-2 possibly attached with mitochondrial ERBB4 and involve viral replication through MAPK signaling pathway. Then, the cell to cell spread action, the replicated SARS-CoV-2 attached to the uninfected AEC2 receptor and then restart macropinocytosis; (3) The part of S2 domain presents peptides into antigen-presenting cells (APC) to modulate host immune system through NFkB- signaling.

In the present study, the highly downregulated gene ERBB4 was selected as a drug target to control SARS-CoV-2 infection. Figure 9 illustrates the hypothesized mechanism action of downregulated ERBB4 in SARS-CoV-2 infected cells. A key receptor tyrosine kinase ERBB4 contains four domains includes multiple furin-like cysteine-rich domains, a tyrosine kinase domain, a phosphatidylinositol-3 kinase binding site, and a PDZ binding domain. Ligands are binding to the ERBB4 which induces different cellular response including alveolarization, alveolar epithelial type 2 cells (AEC2) differentiation, anchoring of cell surface receptor, and morphogenesis via extracellular-signal-regulated kinase pathway ^82, 83^. Downregulation of ERBB4 inhibits regular action namely alveolarization, morphogenesis, and AEC2 differentiation. Many proteolytic events allow for the release of ERBB4 cytoplasmic fragments. The downregulation of ERBB4 by the SARS-CoV-2 in AEC2 cells may induce receptor tyrosine kinase-mediated macropinocytosis for the host cell entry. Besides, activation of macropinocytosis can increase non-specific fluid uptake, the pH in the vesicle is not decreased and, in that scenario, SARS-CoV-2 may be recycled to the cell surface. Interestingly, fluid-phase uptake (macropinocytosis and cell-to-cell spread) was reported in murine coronavirus and SARS-CoV infection ^84– 86^. Both macropinocytosis and PI3K signaling are directly associated with the progression of the number of tumor cells including pancreatic and lung cancer ^87, 88^. Further, growing evidence suggested that PI3K signaling involves the generation of the second messenger lipids and activates Rac-Pak signaling which plays a critical function namely macropinocytosis, mTOR signaling clathrin-mediated endocytosis, and cytoskeletal rearrangements ^89, 90^. A recent systematic review by 16 and his colleagues showed the increased level of biomarkers such as C-reactive protein, serum amyloid A, interleukin-6, lactate dehydrogenase, D-dimer, cardiac troponin and renal biomarkers were higher in severe complicated COVID-19 patients’ plasma and infected lung tissues. Another study reported that low levels of type I and III interferons reduced innate antiviral defenses, which act as driving features of COVID-19 ^91^. In addition, proteome analysis of SARS-CoV-2 infected hosts demonstrated that proteins involved in translation, splicing, carbon metabolism, proteostasis and nucleic acid metabolism could act as a drug target for SARS-CoV-2 ^92^. Moreover, Gordon *et al*. reported 67 druggable targets and repurposed 69 FDA-approved drugs for COVID-19 ^93^. This information supports the technical concept of the present study which enhances drug repurposing strategy and advances on precision-based therapeutics to COVID-19.

In the present study, we repurposed Wortmannin as a candidate drug that can inhibit PI3K signaling and receptor tyrosine kinase (ERBB4) mediated macropinocytosis, to control virus-cell proliferation and may enhance ERBB4 functions namely alveolarization, morphogenesis, and AEC2 differentiation. Wortmannin is a covalent inhibitor of phosphatidylinositol-3-kinase (PI3K) and related enzymes include the mammalian target of rapamycin (mTOR), DNA-dependent protein kinase catalytic subunit (DNA-PKcs), and mitogen-activated protein kinase (MAPK) ^94, 95^. In humans, PI3K activates cell signaling pathways in the head and neck, urinary tract, cervical, ovarian, and lung cancer. Studies reported that the inhibition of PI3K signaling enhances tumor suppression and anti-tumor activity ^96^. Besides, Wortmannin is an extensively used cell biology research includes to inhibit cell proliferation, DNA repair, and receptor-mediated endocytosis ^97, 98^. This report supported that the repurposed Wortmannin may have the potential to control SARS-CoV-2 infection in COVID-19 patients with downregulated expression of receptor tyrosine kinase ERBB4.

## Conclusion

Biomarkers can be used as part of a personalized medicine paradigm to customize treatment to the specific disease characteristics of an individual patient. It can also be used to better understand disease mechanisms and to identify novel disease targets. In the present study, the highly downregulated gene ERBB4 was selected as a drug target and Wortmannin was repurposed as candidate drug to control SARS-CoV-2 infection. Further, this study provides insights on the possible personalized therapeutics for emerging COVID-19.

## Data availability

### Underlying data

Zenodo: Datasets for SARS-CoV-2 drug target and candidate drug identification, http://doi.org/10.5281/zenodo.4458252 ^43^

This project contains the following underlying data:

- List of selected differentially expressed coronavirus infected host genes for protein-protein interaction network construction
- List of protein-protein interaction subnetworks
- List of Walktrap modules and potential drug targets
- Potential Drug Targets and SARS-CoV-2 drug targets docking results
- List of predicted candidate drugs for proposed drug target
- Complete list of DEG’s

Data are available under the terms of the Creative Commons Attribution 4.0 International license (CC-BY 4.0).
