# Supplementary material for: Identifying potential drug targets and candidate drugs for COVID-19: biological networks and structural modeling approaches
Source: F1000Res. 2021 May 17;10:Chem Inf Sci-127. Originally published 2021 Feb 18. [Version 3] doi: 10.12688/f1000research.50850.3 (PMC8080978; doi:10.12688/f1000research.50850.3)
Supplement: Peer review history

# Peer review: Reviewer response for version 3

**DOI:** 10.5256/f1000research.56619.r85487

**Published:** 2021-06-07

**Authors:** Dhurvas C. Dinesh

**Affiliations:** 1 Section of Molecular Biology and Biochemistry, Institute of Organic Chemistry and Biochemistry, Prague, Czech Republic

Many thanks. I approve the manuscript by Selvaraj *et al.* for indexing.

Is the work clearly and accurately presented and does it cite the current literature?

Yes

If applicable, is the statistical analysis and its interpretation appropriate?

Yes

Are all the source data underlying the results available to ensure full reproducibility?

Yes

Is the study design appropriate and is the work technically sound?

Yes

Are the conclusions drawn adequately supported by the results?

Yes

Are sufficient details of methods and analysis provided to allow replication by others?

Yes

Reviewer Expertise:

NA

I confirm that I have read this submission and believe that I have an appropriate level of expertise to confirm that it is of an acceptable scientific standard.

# Peer review: Reviewer response for version 3

**DOI:** 10.5256/f1000research.56619.r85486

**Published:** 2021-05-27

**Authors:** Turanli Beste

**Affiliations:** 1 Department of Bioengineering, Marmara University, Istanbul, Turkey

Thanks to the authors for the revised version.

Is the work clearly and accurately presented and does it cite the current literature?

Yes

If applicable, is the statistical analysis and its interpretation appropriate?

Yes

Are all the source data underlying the results available to ensure full reproducibility?

Yes

Is the study design appropriate and is the work technically sound?

Yes

Are the conclusions drawn adequately supported by the results?

Yes

Are sufficient details of methods and analysis provided to allow replication by others?

Yes

Reviewer Expertise:

Systems Biology; Drug repositioning; Cancer Biomarkers

I confirm that I have read this submission and believe that I have an appropriate level of expertise to confirm that it is of an acceptable scientific standard.

# Peer review: Reviewer response for version 2

**DOI:** 10.5256/f1000research.55646.r82773

**Published:** 2021-04-22

**Authors:** Sugunadevi Sakkiah

**Affiliations:** 1 Division of Bioinformatics and Biostatistics, National Center for Toxicological Research, Jefferson, AR, USA

Accept for indexing in the current form.

Is the work clearly and accurately presented and does it cite the current literature?

Yes

If applicable, is the statistical analysis and its interpretation appropriate?

Yes

Are all the source data underlying the results available to ensure full reproducibility?

Yes

Is the study design appropriate and is the work technically sound?

Yes

Are the conclusions drawn adequately supported by the results?

Yes

Are sufficient details of methods and analysis provided to allow replication by others?

Yes

Reviewer Expertise:

NA

I confirm that I have read this submission and believe that I have an appropriate level of expertise to confirm that it is of an acceptable scientific standard.

# Peer review: Reviewer response for version 2

**DOI:** 10.5256/f1000research.55646.r83072

**Published:** 2021-04-20

**Authors:** Turanli Beste

**Affiliations:** 1 Department of Bioengineering, Marmara University, Istanbul, Turkey

I enjoyed reading the article. Methods are applied precisely and sound reproducible. Results and Discussion parts are written well and well-organized. There are minor comments that need to be addressed.

1. In the DEG list, there is no information about the expression of the ACE2 receptor which is not only an enzyme but also a functional receptor on cell surfaces through which SARS-CoV-2 enters the host cells and is highly expressed in the heart, kidneys, and lungs and shed into the plasma. However, it is not a DEG on your list. Maybe, it is better to provide a complete list and the readers can see the results about other genes as well.
2. "Downregulation of ERBB4 inhibits regular action namely alveolarization, morphogenesis, and AEC2 differentiation" AEC2 or ACE2? It looks like a typing error made in Figure 9 as well as the text. Otherwise, I could not find information on the AEC2 protein.

Is the work clearly and accurately presented and does it cite the current literature?

Yes

If applicable, is the statistical analysis and its interpretation appropriate?

Yes

Are all the source data underlying the results available to ensure full reproducibility?

Yes

Is the study design appropriate and is the work technically sound?

Yes

Are the conclusions drawn adequately supported by the results?

Yes

Are sufficient details of methods and analysis provided to allow replication by others?

Yes

Reviewer Expertise:

Systems Biology; Drug repositioning; Cancer Biomarkers

I confirm that I have read this submission and believe that I have an appropriate level of expertise to confirm that it is of an acceptable scientific standard, however I have significant reservations, as outlined above.

# Response

**Published:** 2021-04-29

**Authors:** Gurudeeban Selvaraj

**Affiliations:** Concordia University, Canada

**Comment 1:** In the DEG list, there is no information about the expression of the ACE2 receptor which is not only an enzyme but also a functional receptor on cell surfaces through which SARS-CoV-2 enters the host cells and is highly expressed in the heart, kidneys, and lungs and shed into the plasma. However, it is not a DEG on your list. Maybe, it is better to provide a complete list and the readers can see the results about other genes as well.

**Author response 1**: Thanks for your appreciation. Yes, the authors agree, the ACE2 receptor is an enzyme and also a cell surface receptor, which permits SARS-CoV-2 to enter the host cells. ACE2 is the key receptor for primary infection. But in the present study, the expression level of ACE2 was found to be less significant (log2FC<1) in the infected cells of all the selected datasets. As per the reviewer's suggestion, the complete list of differentially expressed genes included in the appendix ( https://zenodo.org/badge/DOI/10.5281/zenodo.4726202.svg).

**Comment 2:** "Downregulation of ERBB4 inhibits regular action namely alveolarization, morphogenesis, and AEC2 differentiation" AEC2 or ACE2? It looks like a typing error made in Figure 9 as well as the text. Otherwise, I could not find information on the AEC2 protein.

**Author Response 2:** Thanks. Yes, it is ACE2, the typo is corrected in the revised version.

# Peer review: Reviewer response for version 2

**DOI:** 10.5256/f1000research.55646.r82880

**Published:** 2021-04-12

**Authors:** Dhurvas C. Dinesh

**Affiliations:** 1 Section of Molecular Biology and Biochemistry, Institute of Organic Chemistry and Biochemistry, Prague, Czech Republic

The manuscript which I review entitled “Identifying potential drug targets and candidate drugs for COVID-19: biological networks and structural modeling approaches [version 2; peer review: 1 approved with reservations]” performed by Selvaraj, et al. seems an interesting piece of work, and well written. 

 Reviewer recommendation: Minor Revision.

1. Based on the results of PPIs the authors have selected about ten proteins as protein drug targets, and are highly expressed after Co-V infection, is this protein expression similar to CoV-2 infection?
2. Several reports have been reported that ACE2 is one of the key proteins involved in viral entry and it shows interaction with viral protein. In this from the PPI analysis it was noticed that the expression of ACE2 after CoV is not shown in figure 2. How was ACE2 expression not in the PPI interaction analysis?
3. The authors have mentioned that the miRNA data set of differentially expressed CoV-HGs was retrieved for this study, but in the analysis PDT, it was mentioned as SARS-CoV-2, to make it clear. 
4. Based on the protein-protein affinity the authors have concluded ERBB4 has high binding affinity and also it was taken for molecular simulation studies, but in the table 3 it was noticed that KLHL12 has more or less similar binding affinity and also has more binding affinity with MLpro than ERBB4, on what basis have the authors selected ERBB4 as a drug target for further studies?

Is the work clearly and accurately presented and does it cite the current literature?

Yes

If applicable, is the statistical analysis and its interpretation appropriate?

Yes

Are all the source data underlying the results available to ensure full reproducibility?

Yes

Is the study design appropriate and is the work technically sound?

Yes

Are the conclusions drawn adequately supported by the results?

Yes

Are sufficient details of methods and analysis provided to allow replication by others?

Yes

Reviewer Expertise:

Virology

I confirm that I have read this submission and believe that I have an appropriate level of expertise to confirm that it is of an acceptable scientific standard, however I have significant reservations, as outlined above.

# Response

**Published:** 2021-04-29

**Authors:** Gurudeeban Selvaraj

**Affiliations:** Concordia University, Canada

**Comment 1:** Based on the results of PPIs the authors have selected about ten proteins as protein drug targets, and are highly expressed after Co-V infection, is this protein expression similar to CoV-2 infection?

**Author Response 1:** Thanks for the question. All the selected datasets contain control and CoV infected human lung epithelial cells samples. In earlier days the inadequate information of the CoV-2 genomic datasets in public databases (i.e. NCBI), the authors have selected CoV samples (i.e. CoV-1 genomic information is more than 96% similar to CoV-2). Further, the expression pattern was verified with protein-protein docking of identified targets with SARS-CoV-2 drug targets (i.e. 3cLpro, N-pro, and SRBD).

**Comment 2:** Several reports have been reported that ACE2 is one of the key proteins involved in viral entry and it shows interaction with viral protein. In this from the PPI analysis, it was noticed that the expression of ACE2 after CoV is not shown in figure 2. How was ACE2 expression not in the PPI interaction analysis?

**Author Response 2:** Thanks for the good question. ACE2 is one of the key receptors for primary infection. But, in the PPI network analysis results of the present study, the expression level of ACE2 was found to be less significant (log2FC<1) in the infected cells of the selected datasets. Thus, the PPI interaction analysis excludes ACE2. In addition, the complete list of differentially expressed genes included in the appendix ( https://zenodo.org/badge/DOI/10.5281/zenodo.4726202.svg).

**Comment 3:** The authors have mentioned that the miRNA data set of differentially expressed CoV-HGs was retrieved for this study, but in the analysis PDT, it was mentioned as SARS-CoV-2, to make it clear.

**Author Response 3:** Thanks. Yes, all the identified PDTs from the CoV-HGs cross-verified with SARS-CoV-2 drug targets (i.e. 3cLpro, N-pro, and SRBD) using protein-protein docking to confirm the interaction pattern. Based on the protein-protein docking results, it is described as PDTs of SARS-CoV-2.

**Comment 4:** Based on the protein-protein affinity the authors have concluded ERBB4 has high binding affinity and also it was taken for molecular simulation studies, but in table 3 it was noticed that KLHL12 has a more or less similar binding affinity and also has more binding affinity with MLpro than ERBB4, on what basis have the authors selected ERBB4 as a drug target for further studies?

**Author Response 4:** Thanks. Yes, the binding affinity of ERBB4 and KLHL12 with SARS-CoV-2 targets (i.e. 3cLpro, N-pro, and SRBD) was almost similar, but both proteins differ in the level of expression in infected cells. In general, the potential drug target selection is based on the log2 fold change (i.e. -2>log2FC<2). In the present study, ERBB4 is highly under-expressed (i.e. log2FC=-2.052) compared to other identified drug targets. Thus, the authors have chosen ERBB4 as a potential drug target for further study.

# Peer review: Reviewer response for version 1

**DOI:** 10.5256/f1000research.53939.r79888

**Published:** 2021-03-22

**Authors:** Sugunadevi Sakkiah

**Affiliations:** 1 Division of Bioinformatics and Biostatistics, National Center for Toxicological Research, Jefferson, AR, USA

The authors put a great effect to identify the potent target and candidate drugs for COVID-19 applying biological networks and structural modeling approaches. The authors should address the below comments before the indexing of this paper:

 Minor revision:

1. In the introduction, Ref. 16, the authors should mention the authors' name.
2. In methods, data collection, the authors should be clear whether they selected all CoV infected samples or only CoV2 infected samples?
3. In Figure 1, SARS-CoV means 1 and 2 or only 2?
4. Any particular reason for the authors setting the 303K in molecular dynamics simulations?

Is the work clearly and accurately presented and does it cite the current literature?

Yes

If applicable, is the statistical analysis and its interpretation appropriate?

Yes

Are all the source data underlying the results available to ensure full reproducibility?

Yes

Is the study design appropriate and is the work technically sound?

Yes

Are the conclusions drawn adequately supported by the results?

Yes

Are sufficient details of methods and analysis provided to allow replication by others?

Yes

Reviewer Expertise:

Cheminformatics, Endocrine disrupting chemicals

I confirm that I have read this submission and believe that I have an appropriate level of expertise to confirm that it is of an acceptable scientific standard, however I have significant reservations, as outlined above.

# Response

**Published:** 2021-03-22

**Authors:** Gurudeeban Selvaraj

**Affiliations:** Concordia University, Canada

**Comment 1:** In the introduction, Ref. 16, the authors should mention the authors' name.

**Author Response 1:** Thanks for your suggestion. The author name is included in the text as follows----A recent systematic review by 16 Kermali and his colleagues showed the increased level of biomarkers such as C-reactive protein, serum amyloid A, interleukin-6, lactate dehydrogenase, D-dimer, cardiac troponin, and renal biomarkers (urea and creatinine) were higher, and low level of lymphocytes and platelet count were recorded in severe complicated COVID-19 patients than non-severe COVID-19 patients’ plasma and infected lung tissues.

**Comment 2:** In methods, data collection, the authors should be clear whether they selected all CoV infected samples or only CoV2 infected samples?

**Author Response 2:** Thanks for the question. All the selected studies contain control and CoV-infected human lung epithelial cells samples. In earlier days the inadequate information of the CoV-2 genomic datasets in Public databases (i.e. NCBI), the authors have selected CoV samples (i.e. CoV-1 genomic information is more than 96% similar to CoV-2).

**Comment 3:** In Figure 1, SARS-CoV means 1 and 2 or only 2?

**Author Response 3:** Thanks for the key question. The initial drug targets were identified from the SARS-CoV-1 whole-genome datasets. Then, the targets verified and validated with RBD-spike receptor binding domain; 3cLpro-3C-like protease; N pro-nucleocapsid protein of SARS-CoV-2 using protein-protein docking. The protein-protein docking results confirm molecular interaction of the identified drug targets with proteins of SARS-CoV-2. Thus, the first box of Figure 1, represents the SARS-CoV or SARS-CoV-1.

**Comment 4:** Any particular reason for the authors setting the 303K in molecular dynamics simulations?

**Author Response 4:** Thanks for the key question. Yes, most computational and experimental studies have focused on room temperature (298K to 303K). The MD simulation input file preparation using the Charmm-gui web-interface was set at 303K as a room temperature (Jo et al., 2008 and Lee et al., 2016). Thus, the authors followed the same.
